# A Preclinical Investigation of Estrogenic Bone Protection in a Hypertensive Rat Model Under Gender-Affirming Hormone Therapy

**DOI:** 10.3390/biology14060650

**Published:** 2025-06-03

**Authors:** Lucas Streckwall, Germán A. Colareda, Daiana Escudero, Romina G. Diaz, Juan M. Fernández

**Affiliations:** 1 Laboratorio de Investigaciones en Osteopatías y Metabolismo Mineral (LIOMM), Facultad de Ciencias Exactas, Universidad Nacional de La Plata (UNLP)-Centro de Investigaciones Científicas (CIC), La Plata B1900, Argentina; lstreckwall@biol.unlp.edu.ar; 2 Farmacología-GFEYEC, Departamento de Ciencias Biológicas, Facultad de Ciencias Exactas, Universidad Nacional de La Plata (UNLP), La Plata B1900, Argentina; gcolareda@biol.unlp.edu.ar; 3 Centro de Investigaciones Cardiovasculares “Dr. Horacio E. Cingolani”, Facultad de Ciencias Médicas, Universidad Nacional de La Plata (UNLP)-Consejo Nacional de Investigaciones Científicas y Técnicas (CONICET), La Plata B1900, Argentina; descudero@med.unlp.edu.ar (D.E.); rgdiaz@med.unlp.edu.ar (R.G.D.)

**Keywords:** bone health, estrogen, gender-affirming care, gender differences, gender-affirming hormone therapy, osteoblast differentiation

## Abstract

The goal of gender-affirming hormone therapy (GAHT) is to align an individual’s physical characteristics with their gender identity by suppressing endogenous sex hormones and replacing them with those consistent with their gender. Transgender women undergoing GAHT are at higher risk of cardiovascular complications, and since clinical evidence suggests that hypertension is associated with increased bone loss, we investigated the effects of estrogen treatment on bone health in a hypertensive transgender animal model. Male spontaneously hypertensive rats were orchiectomized (Orch), and half of them received estrogen treatment (Orch + Es), while a third group remained intact as controls. Bone marrow progenitor cells (BMPCs) were isolated to assess osteogenic potential, and femurs were collected for histological and mechanical analysis. BMPCs from Orch + Es rats exhibited enhanced osteogenic potential compared to those from Orch rats. Histological analysis revealed a higher number of osteocytes and fewer adipocytes in the Orch + Es group. Mechanical testing showed reduced bone strength in Orch rats, which was partially preserved in Orch + Es animals. In conclusion, estrogen administration mitigated the deleterious effects of testosterone depletion on BMPCs and provided protective effects on bone structure and strength in this preclinical model of GAHT in hypertensive rats.

## Simple Summary

Transgender people often undergo gender-affirming hormone therapy (GAHT) to address the incongruence with their gender identity. This treatment adjusts hormone levels and reduces physical traits that do not match a person’s gender identity. While GAHT is important for many transgender people, scientists are still studying how it affects the body. For example, transgender women who take estrogen may have a higher risk of heart problems. Estrogen also affects bone health, but past studies have shown mixed results. It is still unclear whether this therapy helps or harms bones over time. High blood pressure is known to cause more bone loss. So, we studied how estrogen affects bone health in a special animal model designed to reflect transgender people with high blood pressure. Our findings showed that estrogen helped protect bone health, even when testosterone levels were low due to surgery. Specifically, estrogen supported bone-forming cells and reduced damage to the femur, the large bone in the leg. These results suggest that estrogen therapy may help protect bones in certain cases, but more research is needed to understand its long-term effects fully.

## 1. Introduction

Gender incongruence refers to the discomfort that arises from the mismatch between a person’s gender identity and the sex assigned at birth [1]. The goal of gender-affirming hormone therapy (GAHT) is to align the individual’s physical characteristics with their gender identity and can be aimed at either masculinizing or feminizing the body. These treatments typically involve administering specific sex hormones, often alongside surgical procedures to alter primary and secondary sexual characteristics [1].

For transgender women, feminizing hormone therapy generally involves administering estrogen, which may be performed alone or in combination with treatments that reduce endogenous androgen levels [2]. In the past, many treatments were carried out clandestinely without medical supervision, making them unsafe. Today, various standardized GAHT protocols are approved by health systems in several countries to target serum levels of sex steroids to match the levels associated with the individual’s gender identity [3]. However, despite the challenges and limitations in epidemiological studies evaluating GAHT [4], several adverse effects associated with gender transition and treatments have been identified [5].

Among the reported side effects in transgender women are increased risk of myocardial infarction and stroke, as well as elevated triglyceride levels [1,4,6,7]. Before and during GAHT protocols, transgender women should be periodically evaluated for signs of cardiovascular failure, cardiovascular risk factors, liver function, and venous thromboembolic disease, but whether bone health must also be considered is a matter of discussion. Although recent clinical practices include recommendations for osteoporosis screening [8], it is not considered in some countries [3].

Regarding bone health, both suppression and reassignment hormonal therapy were associated with decreasing bone mineral density [9,10]. Estrogen administration can affect bone metabolism since estrogen receptors α and β are expressed in several bone cells such as osteoclasts, osteoblasts, lining cells, and osteocytes [11]. Estrogens directly influence osteoblasts, stimulating the production of markers associated with their differentiation while inhibiting their apoptosis [12,13]. At the same time, estrogens regulate the number and activity of osteoclasts by promoting their apoptosis and thus limiting bone resorption [14]. On the other hand, a decrease in testosterone levels can lead to a reduction in bone mineral density (BMD), as bone tissue also expresses the androgen receptor [15]. Transgender individuals, including those who are gender non-conforming, report higher rates of negative health behaviors and lower bone mineral density (BMD), which may be attributed to inadequate calcium intake and vitamin D deficiency [16,17]. Additionally, they face barriers such as stigma and discrimination that limit their participation in physical activity [16]. Several experts agree on the importance of adaptive nutrition and exercise intervention programs according to gender identity to improve health and well-being in this population [18,19,20,21,22,23]. Another risk factor for bone loss is related to hypertension [24], with World Health Organization (WHO) reports of 1 in 4 men and 1 in 5 women having this condition, without epidemiological data for transgender people [25]. Despite this, bone mineral density can improve with gender-affirming hormone therapy (GAHT). However, the clinical effects of testosterone reduction associated with increased estradiol on bone health are poorly understood. A baseline study on bone densitometry reported T-scores below −2.5 in 16% of trans women [23]. Studies on cisgender adult males suggest that serum estradiol correlates more positively with bone mineral density than testosterone, suggesting that estrogens would preserve bone mineral density in trans women who continue estrogen and antiandrogen regimens [26]. A recent meta-analysis concluded that trans women undergoing GAHT showed an increase in BMD [7]. There are no available data on fractures in trans women. Those who discontinue hormonal treatment after gonadectomy likely have an increased risk of bone loss. Therefore, over time, BMD may decrease again, leading transgender women over 50 years old to have a fracture risk similar to that of cisgender women of the same age [5,16,27,28]. The effects of GAHT on bones are not limited to adults; puberty is a crucial period for bone development, with increased estrogen and androgen levels promoting skeletal development. Initiation of GAHT can lead to increased bone mineral density [27], but the negative impact of prolonged pubertal suppression is not always fully recovered. Interestingly, in transgender girls, changes in lean and fat mass were more pronounced over the three years of puberty suppression, stabilizing in the first year of estradiol treatment, while in transgender boys, the changes were smaller and reversed with the initiation of testosterone, particularly during the first year [28].

Due to differences in GAHT treatments (both clandestine and regulated), lifestyle, age of therapy initiation, and the lack of controlled trials, continuous monitoring of BMD in transgender women is essential [5,29,30,31,32]. Bone health in transgender individuals is an emerging area of research that is receiving increasing attention in the medical literature. As more individuals seek gender-affirming treatments, it is crucial to understand how hormone therapy, especially the use of estrogens, affects BMD and cardiovascular health in this population.

Although the effects of estrogen administration following orchiectomy have been previously studied on the skeletal and cardiovascular systems in the context of prostate cancer treatment, research has been limited to examining mechanical and/or histological aspects, without exploring the molecular effects or cellular behavior that could help to find new therapeutic targets that are still unknown [33,34,35,36,37].

Given that estrogen is a key factor in bone health, particularly after orchiectomy or GAHT, and given the high impact of hypertension in the global population, understanding estrogen’s impact on bone structure and metabolism in transgender women is essential.

In this study, we evaluated for the first time the effect of estrogen administration on bone in a model of transgender hypertensive female rats, providing valuable insights into the impacts of GAHT on bone health.

## 2. Material and Methods

### 2.1. Animal Treatments

All animal procedures were performed in accordance with the Guide for the Care and Use of Laboratory Animals published by the National Research Council, National Academy Press, Washington DC 2010, and approved by the Ethical Committee of Faculty of Medical Sciences of National University of La Plata (#P05-01-2024). Due to their equivalence to an adolescent human skeleton [2], 12 three-month-old male spontaneously hypertensive rats from the Central Animal Facility of Faculty of Medical Sciences of National University of La Plata were housed in a 12 h dark/light cycle, temperature of 25 °C, humidity of 50%, and fed with standard diet ad libitum.

The animals selected for these experiments were separated at the time of weaning into three groups of four animals and placed and kept in three cages (one for each group) for the entire treatment period. Since they have no interaction with females or with other males after gonadectomy or estrogen treatment, it is not possible to establish a reproductive history performance. Bilateral orchiectomy was performed chirurgically by testicle remotion, and animals were kept in their cages with no contact with cisgender females or males. For that reason, neither reproductive history nor hystological analysis of the testicle tissue or sperm count were performed. This is a crucial difference with the treatments based on hormonal inhibition of the endogenous axis (for example with cyproterone) [38].

**Figure 1:**
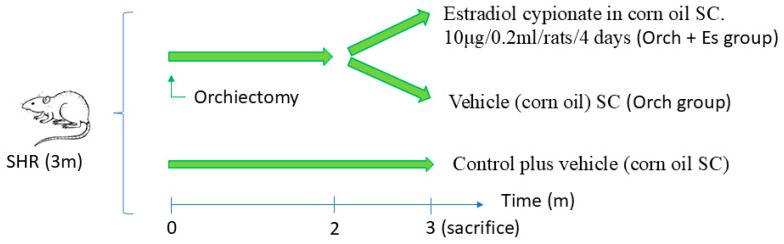
Treatment protocol in spontaneously hypertensive rats (SHRs).

For hormonal treatment protocols, rats were divided into two groups: orchidectomy (n = 8, bilateral orchidectomy performed under inhalatory anesthesia with isofluorane 2% plus intraperitoneal dexamethasone 0.5 mg/kg and tramadol 15 mg/kg) and a Control group (n = 4) without gonadectomy surgery but exposed to the same anesthetic/analgesic procedure. Two months after orchidectomy, for one month, one subgroup of these rats received estrogen therapy subcutaneously with estradiol cypionate in corn oil (10 μg/0.2 mL/rat) every 4 days, mimicking the estrogen peak of the estrous cycle (Orch + Es; n = 4) [39]; the other was left as a control of the surgery (Orch; n = 4) (Figure 1). Systolic arterial pressure was monitored at the beginning and the end of treatment using the non-invasive tail cuff method [40]. At the time of sacrifice, rats were anesthetized with tramadol via subcutaneous (30 mg/Kg) and urethane via intraperitoneal (1 g/kg) until deep anesthesia was reached; they were then killed by extracting the heart. Immediately after sacrifice, blood samples were extracted and centrifuged (20,000× *g* for 20 min) to perform testosterone and estradiol concentration analysis in plasma by ELISA kits (BIOLINE BPR08, Beckman Coulter, Fullerton, CA, USA).

### 2.2. Cell Cultures

#### 2.2.1. Bone Marrow Progenitor Cell Isolation and Incubation

Bone marrow progenitor cells (BMPCs) were isolated from humeri. After the sacrifice of rats, humeri were dissected and BMPCs were obtained by flushing the diaphysal medullary canal with Dulbecco’s modified essential medium (DMEM) (Gibco, ThermoFisher, Waltham, MA, USA) under sterile conditions. The resulting suspension was seeded in a 25 cm^2^ tissue culture flask and incubated with DMEM supplemented by penicillin (100 UI/mL), streptomycin (100 μg/mL), and 10% fetal bovine serum (FBS) (Internegocios S.A., Mercedes, Argentina) at 37 °C in a humidified atmosphere with 5% CO_2_—95% air [41]. After 24 h, the culture medium was changed to remove non-adherent cells. When cells reached confluence, they were resuspended with trypsin/EDTA and replated as indicated.

#### 2.2.2. Osteogenic Differentiation of BMPCs

For osteogenic differentiation studies, 10^4^ BMPCs per well were plated in 24-well plates with DMEM—10% FBS at 37 °C with 5% CO_2_. When cells reached 70% confluence, the culture medium was changed for osteogenic media (DMEM + 10% FBS + 25 μg/mL ascorbic acid + 5 mmol/L sodium β-glycerolphosphate), which was replaced twice a week. To confirm the osteogenic differentiation of cells, lineage-specific markers were evaluated: alkaline phosphatase-specific activity and type 1 collagen deposition after 14-day cultures in osteogenic media.

After 14 days in osteogenic media, cell monolayers were washed with phosphate-buffered saline (PBS) and lysed with 250 μL 0.1% Triton-X100. An aliquot of each extract was used to evaluate alkaline phosphatase activity (ALP) by hydrolysis of p-nitro phenylphosphate (p-NPP) to p-nitrophenol (p-NP) at 37 °C. The absorbance of p-NP was measured at 405 nm [41]. Another aliquot of each extract was used for protein determination by Bradford’s technique [42]. Type 1 collagen production was evaluated by Sirius Red staining. Briefly, cell monolayers were washed with PBS, fixed with Bouin’s solution, and stained with Sirius Red dye for 1 h. The stained material was dissolved in 1 mL of 0.1 N sodium hydroxide and the absorbance of the solution was measured at 550 nm [41].

#### 2.2.3. PCR Evaluation of Osteogenic Markers

For PCR studies, total RNA was isolated from cell cultures submitted to osteogenic induction for 14 days [41] using QUICKZOL (Kalium Technologies, Buenos Aires, Argentina) reagent according to the manufacturer’s instructions. Expression of osteogenic markers such as collagen type 1 alpha 1 chain (*Col1a1*), *osteocalcin* (*OC*), *receptor activator of nuclear factor-κB-ligand* (*RANK-L*), and *osteoprotegerin* (*OPG*) were evaluated by semi-quantitative RT-PCR using Moloney Murine Leukemia Virus Reverse Transcriptase (MMLV-RT) (Invitrogen, ThermoFisher, USA). The expression of all markers was normalized to that of *β-actin* (housekeeping gene), and the ratio *RANK-L*/*OPG* was calculated as an osteoclast-inducing profile. Specific primers (Table 1) were designed by NCBI sequence data using CLC Genomic Workbench software Premium (QIAGEN CLC) and synthesized by Macrogen (Seoul, Republic of Korea). After separation of RT-PCR products by agarose electrophoresis and their visualization with GelGreen, the intensity of bands was quantified using ImageJ software 1.53k.

### 2.3. Bone Histomorphometric Examination

Right femora were processed for quantitative histomorphometric analysis. Dissected bones were cleaned of soft tissue, fixed in 10% formalin, and decalcified in 10% EDTA. Then, they were embedded in paraffin, and 5 μm sections of proximal femur were obtained using an RMT-20 Type Erma microtome (TechLabs, Ambala Cantt, India). Sections were either stained with haematoxylin–eosin (H&E). Pictures were taken with a Nikon Coolpix 4500 digital camera on an Eclipse E400 (Nikon, Tokyo, Japan) microscope and analyzed using the ImageJ Program with a microscope scale plugin. Microarchitecture of proximal secondary spongiosa was evaluated: trabecular bone area (as %), trabecular bone osteocytic density (as osteocytes/mm^2^), bone marrow adipocytic density (as adipocytes/mm^2^) [41,43].

### 2.4. Mechanical Three-Point Bending Analysis

A three-point bending test was performed on left femora at 50% of total bone length, using an electromechanical testing machine (Digimess TC500, Digimess, Buenos Aires, Argentina) with a load cell of 500-N capacity (Interface, Scottsdale, AZ, USA) at room temperature, as well as a 20 mm length span (L) and a loading speed of 5 mm/s. Load F (applied in an anterior–posterior direction) and displacement D were recorded until rupture. The maximum load supported by the bone before rupture (FMax) was regarded as its ultimate strength. Data for each sample was used to obtain its stress–strain curve and the following whole-bone mechanical properties were determined: Maximum elastic deflection (yield point displacement Dy) and maximum load supported elastically at yield point (Fy) were used to calculate stiffness at yield point (Fy/Dy) for each sample. Work-to-fracture was defined as the amount of energy the bone absorbed while deforming (Eabs) and determined as the area under the stress–strain curve. Eabs was calculated for the elastic (pre-yield), plastic (post-yield), and total periods of bone deformation (E-Eabs, P-Eabs, and T-Eabs) [43,44,45].

Due to the bone segments between the supports exhibiting characteristics similar to hollow, elliptical cylinders, measuring the horizontal and vertical external (H and V) and internal (h and v) diameters of the fracture sections through micromorphometry allowed us to determine the following geometric and intrinsic material properties [46,47]: Cross-sectional cortical bone area (mm^2^) was defined as 3.14 (HV − hv)/4. Second moment of inertia of cortical bone relation to the antero-posterior bending axis (mm^4^), or Ix, was defined as as 3.14 (H^3^V − h^3^v)/64. To calculate the resistance and intrinsic stiffness of the material, yield stress (N/mm^2^) was defined as FyLB/8Ix and elastic modulus (N/mm^2^) as FyL^3^/48DyIx (N/mm^2^), represented by the slope of the stress–strain curve within the elastic region, respectively.

### 2.5. Statistical Analysis

Results are expressed as mean ± SEM and, unless indicated otherwise, were obtained from two separate experiments performed in triplicate. A one-sample *t*-test was chosen to evaluate discrimination capacity in the choice phase. Differences between groups were assessed by one-way ANOVA with Tukey post hoc test using GraphPad InStat version 3.00 (GraphPad Software). *p* < 0.05 was considered significant for all statistical analyses.

## 3. Results

### 3.1. Blood Pressure and Hormonal Profile Analysis

**Figure 2:**
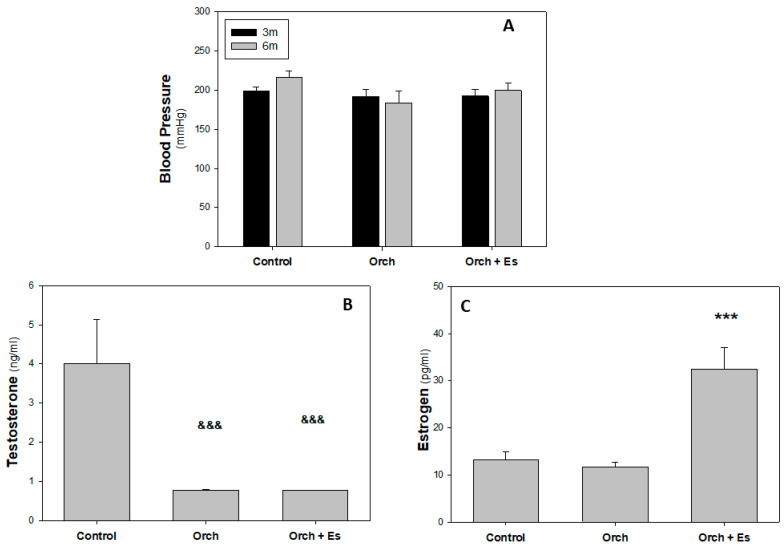
Blood pressure and hormonal profile analysis: Blood pressure in 3-month-old animals (before treatment) and 6-month-old animals (after treatment), along with their serum hormone profiles at the time of sacrifice. (**A**). Systolic blood pressure of 3-month-old (3 m) and 6-month-old (6 m) animals. (**B**). Serum Testosterone levels (ng/mL), (**C**). Serum Estradiol levels (pg/mL). &&&: *p* < 0.001 with respect to Control group. ***: *p* < 0.001 with respect to the Control and Orch groups. Data are expressed as mean ± SEM.

Blood pressure measurements were obtained at two time points: at 3 months of age—immediately before animal allocation into experimental groups and surgical procedures—and at 6 months of age (at the end of the study). As shown in Figure 2A, no significant differences in systolic blood pressure were observed among the different groups (approximately 200 mmHg). Immediately after sacrifice, blood was collected from the animals to determine serum concentrations of testosterone and estradiol hormones. Figure 2B shows that testosterone levels significantly decrease in orchiectomized rats (82.5% lower compared to the Control group). On the other hand (Figure 2C), we found that estradiol levels in rats are significantly elevated only in the serum of the Orch + Es group compared to the Control and in the Orch group (nearly threefold), thus evidencing the efficacy of the estrogen treatment scheme.

### 3.2. Ex Vivo Studies of BMPCs from Orchiectomized Rats Treated with Estradiol

**Figure 3:**
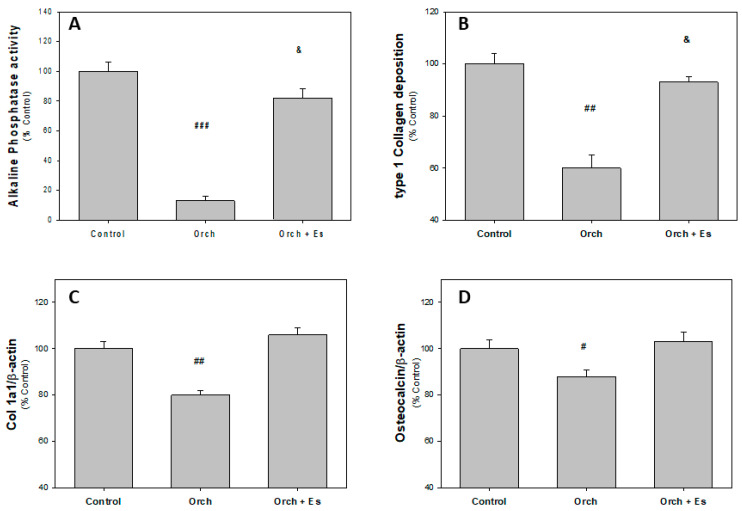
Osteoblastic activity markers of BMPCs after 14 days in osteogenic media: (**A**). Alkaline phosphatase activity, (**B**). type 1 collagen deposition, (**C**). *Collagen 1a1* expression, and (**D**). *osteocalcin* expression. #: *p* < 0.05 vs. Control and Orch + Es groups; ##: *p* < 0.01 vs. Control and Orch + Es groups; ###: *p* < 0.001 vs. Control and Orch + Es groups; &: *p* < 0.05 with respect to the Control group. Data are expressed as mean ± SEM.

After the sacrifice of the animals, the humeri were dissected to obtain BMPCs and we evaluated their capacity to differentiate into osteoblasts (Ob) in an osteogenic medium after 14 days. In Figure 3A, we found that BMPCs derived from Orch rats exhibited lower alkaline phosphatase activity compared to BMPCs from the Control group rats. Regarding BMPCs from the Orch + Es group, they showed higher alkaline phosphatase activity than cells from the Orch group but still lower than cells from the Control group. A similar effect was observed when evaluating type 1 collagen by the cells (Figure 3B). In addition to evaluating these two parameters of osteoblastic activity, we also assessed the expression of several marker genes of the osteoblastic lineage, using *β-actin* as a housekeeping gene since the expression of this gene has not varied between the groups. When evaluating the expression of the collagen gene, we found that BMPCs from Orch rats had lower expression of this gene compared to the Control group. Interestingly, unlike the deposition of type 1 collagen, estradiol administration led to an increase in the expression of this gene in the Orch + Es group, reaching similar levels to those found in the Control group (Figure 3C). In Figure 3D, it can be seen that BMPCs from Orch rats exhibited lower *osteocalcin* (*OC*) gene expression compared to the Control group, an effect that was completely reversed with estradiol administration.

**Figure 4:**
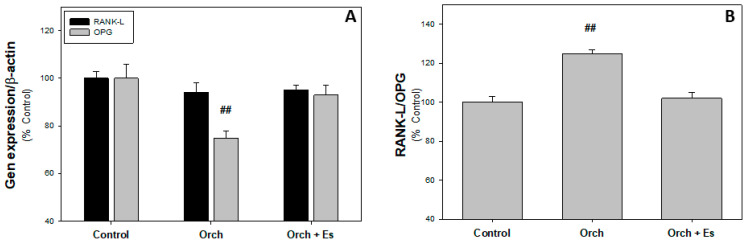
Osteoclastoinductor profile of BMPCs: (**A**). *RANKL* and *OPG* expression. (**B**). *RANK-L*/*OPG* ratio. ##: *p* < 0.01 vs. Control and Orch + Es groups. Data are expressed as mean ± SEM.

In Figure 4A, it can be observed that there are no significant changes in *RANK-L* expression between groups; however, the level of *OPG* expression is decreased in cells from Orch rats and estradiol administration reversed this effect. In Figure 4B, we found that orchidectomy increased the *RANK-L*/*OPG* ratio compared to cells from the Control group, an effect that was reversed when estrogen therapy was administered.

### 3.3. Protective Effect of Estradiol on Bone Microarchitecture

**Figure 5:**
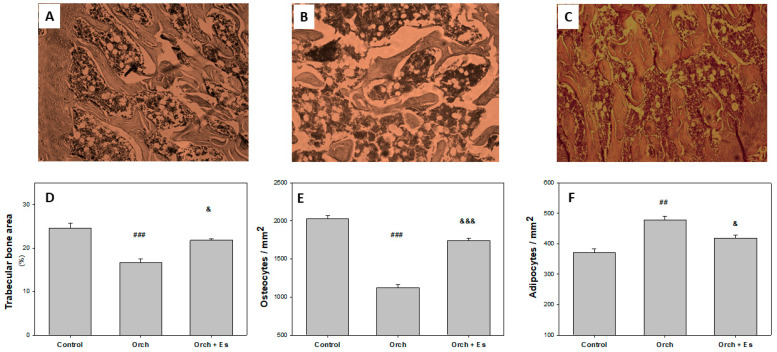
Histological parameters: Representative image of the femoral head (400× magnification). (**A**). Control, (**B**). Orch, (**C**). Orch + Es. (**D**). Percentage of trabecular area. (**E**). % of osteocytes in trabeculae. (**F**). % of adipocytes in bone marrow. ##: *p* < 0.01 vs. the Control and Orch + Es groups; ###: *p* < 0.001 vs. the Control and Orch + Es groups; &: *p* < 0.05 respect to the Control group; &&&: *p* < 0.001 respect to the Control group. Data are expressed as mean ± SEM.

After assessing how estradiol reversed the effects of testosterone deficiency on BMPC osteogenic capacity, we performed histological studies to evaluate its impact on bone microarchitecture. Figure 5A–C shows 400× magnification images of the femoral head beneath the growth plate, stained with Hematoxylin and Eosin, from the Control, Orch, and Orch + Es groups. Analyzing the histological sections, we found that orchiectomy caused a significant reduction in trabecular area compared to the Control (Figure 5D). Although the Orch + Es group also showed a decrease in trabecular area compared to the Control group, it was significantly higher than the Orch group. A similar effect was observed for the percentage of osteocytes (Figure 5E). Interestingly, when evaluating marrow adiposity, we found that the number of adipocytes in the bone marrow was higher in the bones from Orch rats compared to the Control group, while estrogen administration partially reversed this increase (Figure 5F).

### 3.4. Effect of Estradiol on the Biomechanical Behavior of the Femur

**Figure 6:**
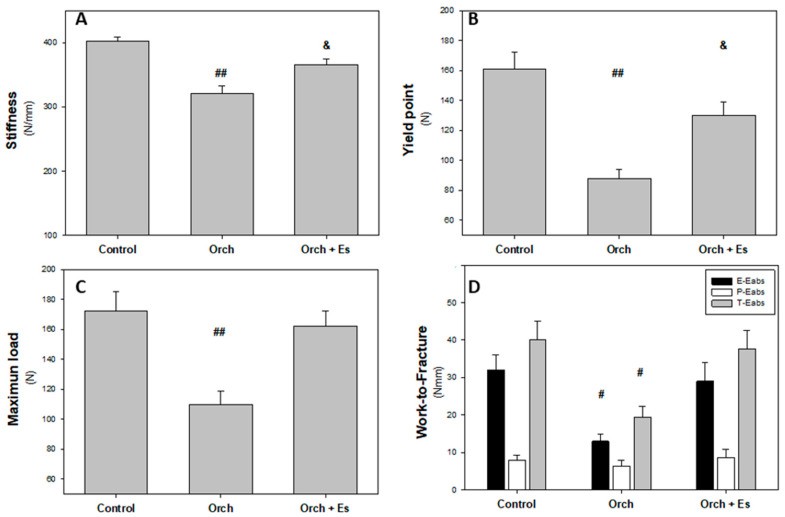
Biomechanical behavior of whole femurs: (**A**). Stiffness (N/mm), (**B**). yield point (N), (**C**). maximum load (N), and (**D**). work-to-fracture and in elastic and plastic regions (Eabs, Nmm). #: *p* < 0.05 vs. Control and Orch + Es groups; ##: *p* < 0.01 vs. Control and Orch + Es groups; &: *p* < 0.05 respect to Control group. Data are expressed as mean ± SEM.

Following dissection, three-point bending tests were performed at the mid-diaphysis of the femur. These tests generated force–displacement curves, which were used to assess the mechanical properties of the cortical bone along the entire femur. The results of these evaluations are presented in Figure 6. As shown in Figure 6A, stiffness was significantly reduced following orchiectomy, an effect that was partially reversed by estrogen administration. In Figure 6B, the yield point was also lower in femurs from the orchiectomized group compared to the Control group. Similar to the results observed for stiffness, estradiol treatment partially restored this parameter. Figure 6C displays the maximum load supported by the femur before fracture. Testosterone deficiency caused a significant reduction in this value; however, estradiol administration fully reversed the effect, restoring maximum load capacity to Control levels. Finally, Figure 6D shows the total energy absorbed (T-Eabs) by the femurs before the fracture. The orchiectomized group exhibited a notable decrease in energy absorption compared to the Control group, but this was effectively reversed by estradiol treatment.

**Figure 7:**
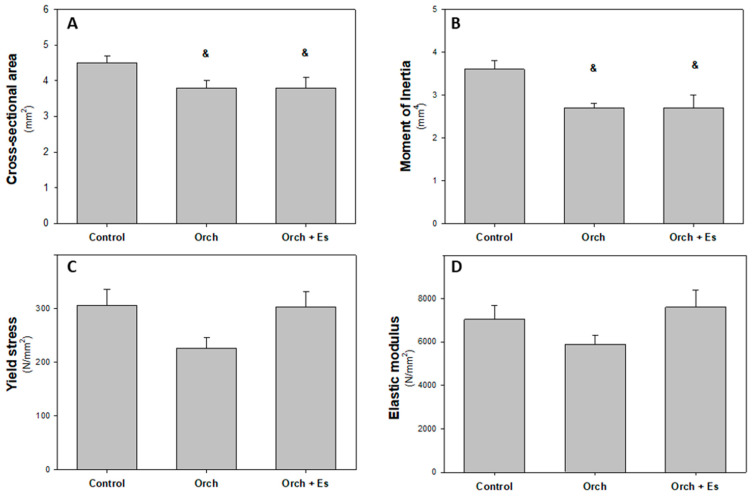
Femur diaphyseal geometric and mechanical intrinsic properties: (**A**). Cross-sectional cortical bone area (mm^2^), (**B**). second moment of inertia of cortical bone relation to the antero-posterior bending axis, Ix (mm^4^), (**C**). yield stress (N/mm^2^), and (**D**). elastic modulus (N/mm^2^). &: *p* < 0.05 respect to the Control group. Data are expressed as mean ± SEM.

In Figure 7, the results of the geometric and intrinsic material properties of bone tissue are shown. In Figure 7A, we can see that orchiectomy resulted in a decrease in cortical bone area, an effect that was not reversed by estradiol administration. The same is observed with the moment of inertia (Figure 7B). However, there were no significant differences in the yield stress (Figure 7C) or the elastic modulus (Figure 7D) of the femurs between the groups.

## 4. Discussion

Gender-affirming hormone therapy (GAHT) consists of protocols that include hormonal treatments used by transgender individuals to modify their secondary sexual characteristics assigned at birth. However, the side effects of these therapies across different age groups of transgender individuals are not yet fully understood [1,4,30,31,47]. On healthy bone, the estrogens can stimulate osteoblasts, promoting bone formation, but a decrease in testosterone, common in transgender women, can lead to lower bone mineral density (BMD). Therefore, animal model studies are becoming increasingly necessary to elucidate the mechanisms involved in bone changes [2,47]. Moreover, as comorbidities could be a common factor in transgender health, it was demonstrated that high blood pressure caused a marked reduction in bone mineral density in both humans and rats [25,48,49]. By comparing the systolic blood pressure values obtained in our study (approximately 200 mmHg) to those reported in the literature for normotensive control Wistar–Kyoto rats, assessed using non-invasive methods (121.2 ± 15.66 mmHg), we confirmed that the rats used in the study were hypertensive [50]. Interestingly, we found no significant differences in blood pressure between the groups or across the two time points. In this work, we studied the effects of estrogen administration in orchidectomized male rats on the BMPC capacity for differentiation to osteoblastic cells in culture. Therefore, we studied microarchitecture bone effects and the whole-bone mechanical properties. The assay of both hormones is crucial for characterizing the model, as feminizing GAHT may include or exclude the use of testosterone production inhibitors, such as gonadotropin-releasing hormone (GnRH) agonists or testosterone effect inhibitors like cyproterone acetate [5]. However, it has been shown that GnRH analogs cause bone loss in young transgender individuals [15], while the health implications of cyproterone are still debated [5,30]. Venkatesh and colleagues administered estradiol to male C57BL/6J mice at concentrations equivalent to those found during the estrous cycle of female mice; however, testosterone levels in these mice did not decrease significantly compared to untreated male mice after 12 weeks of treatment [51]. In our transgender animal hypertensive model, we chose to perform orchiectomy to achieve low testosterone levels to evaluate only the effect of estrogen therapy alone on bone without interference from testosterone-inhibiting drugs.

Osteocalcin (OC) is the most abundant non-collagenous protein in the bone extracellular matrix and serves as a reliable marker of osteoblast activity and bone formation [52,53,54]. In our study, we observed a reduction in OC gene expression, as well as decreases in both type I collagen deposition and gene expression; in alkaline phosphatase (ALP) activity, a key enzyme involved in osteoblast-mediated mineralization, was observed in cells derived from orchiectomized rats. Notably, these alterations in osteogenic potential were reversed following estradiol treatment.

The literature presents mixed findings regarding the effects of gender-affirming hormone therapy (GATH) on OC levels and other bone health markers. For instance, van Caenegem et al. reported decreased serum OC levels in transgender women after two years of GATH [55], whereas Delgado-Ruiz et al. found no significant changes in serum OC levels or ALP activity post-GATH [56]. Similarly, Gava et al. observed no changes in serum OC and ALP after one year of therapy [57], in contrast with findings by Vlot et al., who reported a decrease in serum ALP in transgender women after one year of GATH [10,58].

Given OC’s hormonal role in multiple tissues, including promotion of insulin secretion from the pancreas, stimulation of adiponectin production in adipose tissue, enhancement of glucose metabolism in skeletal muscle, support of cognitive function in the brain, and stimulation of testosterone production by Leydig cells [53], its monitoring in individuals undergoing GATH is widely recommended by researchers worldwide [5,29,30,31]. Although our model involved orchiectomized rats, further research is necessary to understand how OC and other bone turnover markers change in transgender women, particularly those who discontinue hormone therapy after surgery [32].

Osteoclasts, the bone-resorbing cells, are derived from colony-forming unit–granulocyte macrophage (CFU-GM) precursors. These precursors express the receptor activator of nuclear factor κB (RANK) on their surface. Upon binding to its ligand RANK-L, produced by osteoblasts, RANK activates signaling pathways that promote osteoclast differentiation and survival. Osteoprotegerin (OPG), also secreted by osteoblasts, acts as a decoy receptor by binding RANK-L, thereby preventing its interaction with RANK and regulating osteoclast activity [59]. Rather than analyzing RANK-L and OPG independently, the RANK-L/OPG ratio is often used as a more accurate indicator of a cell’s osteoclastogenic potential [60].

In our study, osteoblasts derived from orchiectomized rats exhibited an increased capacity to stimulate CFU-GM differentiation into osteoclasts, primarily due to reduced OPG expression, an effect that was mitigated by estrogen treatment. Although specific data on RANK-L and OPG expression in the transgender population are currently limited, our findings are consistent with related research. For example, Chen et al. (2004) demonstrated that testosterone increases OPG expression, but not RANK-L, in both MC3T3-E1 and mouse osteoblasts [61]. In line with this, osteoblasts from orchiectomized rats (lacking testosterone) in our study showed decreased OPG expression.

Furthermore, research by Bord et al. found that estrogen treatment in primary human osteoblast cultures led to elevated levels of both RANK-L and OPG at 24 h; however, after 48 h, only OPG levels remained elevated while RANK-L returned to baseline [62]. Similarly, we observed increased OPG expression in osteoblasts from orchiectomized rats after one month of estrogen treatment, while RANK-L expression remained unchanged.

After evaluating how estradiol administration reversed the deleterious effects (some partially, others totally) on the osteogenic capacity of BMPCs caused by testosterone deficiency, we conducted histological studies to understand the impact on bone microarchitecture. Orchiectomy induced detrimental changes in bone microarchitecture, as evidenced by a significant decrease in trabecular area, a reduction in the number of osteocytes, and an increase in bone marrow adiposity. However, estrogen administration partially reversed these effects, suggesting a protective role of the hormone on bone tissue under androgen-deprived conditions. Recently, Stratos and colleagues demonstrated through μ-CT a decrease in trabecular bone in the tibia of 12-month-old male Wistar rats after 8 and 12 weeks of orchiectomy. This decrease was prevented with a running training protocol, and although Stratos’s experiments were not conducted in a transgender context, they showed that exercise is closely linked to bone metabolism in orchiectomized rats [63]. Previously, Dobrolinska and colleagues demonstrated a decrease in bone mineral density in the hip of trans women after 10 and 15 months of gonadectomy and hormone therapy [64]. Recently, Motta and colleagues demonstrated a decrease in bone mineral density in transgender women following gender confirmation surgery, partly due to non-compliance with GAHT [22]. Snyder suggests that some bone effects of testosterone are mediated by its conversion to estradiol, catalyzed by CYP19 (gene encoding aromatase); therefore, the effects of GATH could depend on the antiandrogen used [65]. Our model is similar to treatments with the GnRH agonist, where the testosterone levels are lower and the osteoporotic risk is higher. The changes that could occur with androgen receptor antagonists such as cyproterone acetate could differ from our results since conversion to estrogens can occur. Interestingly, it has been shown that estrogen action is critical to estrogen receptor α in osteocytes in female mice, but not in males [66].

After evaluating the impact of hormone therapy on the osteogenic potential of BMPCs and bone microarchitecture, it was also necessary to investigate whether these cellular and structural changes translated into functional improvements at the whole-bone level. In this context, mechanical testing of the entire femur via three-point bending at the mid-diaphysis provided key insights. The resulting force–displacement curves allowed for the assessment of cortical bone strength, offering a comprehensive understanding of how hormonal modulation affects bone integrity beyond the microscopic scale [43,44,45]. Stiffness, which can be interpreted as the femur’s elasticity, refers to the ability of the bone to undergo reversible deformations without resulting in fractures. Orchiectomy led to a decrease in elasticity, resulting in a stiffer bone with a reduced capacity for elastic deformation. Estradiol therapy significantly increased elasticity, although values remained lower compared to femurs from the Control group rats. After a certain applied load (the yield point), the mechanical behavior of the femur transitions from elastic to plastic. Beyond the yield point, cracks begin to accumulate, and deformation becomes irreversible. In our study, the yield point was lower in the femurs following orchiectomy compared to the control group. Similar to stiffness, estradiol therapy partially reversed this effect, improving the yield point, though it did not fully restore it to control levels. Furthermore, the maximum load supported by the femur before fracture was also evaluated. This parameter is critical for understanding the bone’s ability to withstand mechanical stress before failure. In our study, the orchiectomized rats exhibited a significant reduction in the maximum load-bearing capacity, which reflects compromised bone strength. Estradiol therapy, however, was able to partially restore this property, suggesting that hormone therapy can improve the bone’s overall structural integrity, though it might not fully recover the baseline strength of healthy, intact bone. Work-to-fracture results from the combination of stiffness, yield point, and maximum load [41]. In our case, the observed difference is attributed to a decrease in the energy absorbed in the elastic region (E-Eabs) of the curve (area under the curve before the yield point), rather than in the plastic region (P-Eabs). This suggests that testosterone depletion reduces the bone’s ability to disperse energy in the cortical bone of the femoral diaphysis up to the yield point, potentially due to defects in the collagen network and/or hydroxyapatite crystals [43,67]. In 1999, Prakasan and colleagues demonstrated in Wistar rats that orchiectomy increased porosity in the intracortical region of the femur and the rate of periosteal bone formation [68]. Recently, Stratos and colleagues found reduced mechanical properties in the tibiae of orchiectomized rats after 8 and 12 weeks of castration, with these effects being prevented by exercise [61]. In a transgender context, it is important to consider lifestyle factors before starting GAHT, as transgender women often have decreased bone mineral density before therapy, partly due to a lack of physical activity [69]. However, Deckard and colleagues showed in C57BL/6 mice that a lack of estrogen in ovariectomized mice resulted in reduced maximum load supported and less energy absorbed in the elastic region, along with increased stiffness [70]. In 2016, Azboy and colleagues demonstrated that estrogen administration in Sprague–Dawley rats partially reversed the increased stiffness of femurs caused by ovariectomy [71]. Interestingly, Venkatesh and colleagues found increased mechanical behavior parameters in femurs of C57BL/6J mice without orchiectomy and with supra-physiological estradiol administration [51].

The mechanical properties of bone depend on two main factors: geometry (bone architecture) and material properties (bone tissue). These properties determine the overall mechanical effectiveness of bone [46]. Although orchiectomy with or without estradiol administration did not affect the yield point or elastic modulus, it did cause changes in the spatial distribution of the bone. This alteration in the distribution of bone material negatively affected the overall mechanical properties of the bone, making it less effective at resisting loads, which translates into a deterioration in its mechanical performance [46,47]. Osteocyte-ERβ (Ot-ERβ) plays a crucial role in the regulation of bone morphology, particularly in the cortical bone of male mice. In young male mice, Ot-ERβ is essential for maintaining trabecular bone mass, but as the mice reach maturity, Ot-ERβ also exerts a protective role in both cortical and trabecular bones [72,73]. The deletion of Ot-ERβ affects the response of cortical bone to mechanical loading applied to the tibia, as this receptor is required to mediate cortical bone mechanoadaptation in males throughout their lifespan. In contrast, Ot-ERβ does not significantly impact trabecular bone mechanoadaptation. Additionally, estrogen signaling mediated by Ot-ERβ during puberty plays a key role in skeletal growth in males, contributing to the radial expansion of cortical bone, while in females, it regulates endocortical resorption and periosteal expansion [72,73]. Although no effects were observed on the evaluated geometric parameters, long-term treatment studies are needed in our experimental model.

One of our limitations was the selection of the groups, since the present work aims to characterize the role of estrogen therapy within the framework of gender-affirming hormone therapy in a hypertensive model. Hypertensive male controls were used as the control group to evaluate the effects, given that hormonal changes could modify the epigenetics of that group. A limitation of our results could be the lack of comparison with cisgender females under the same conditions. New experiments are currently being carried out in this regard.

## 5. Conclusions

Our simplified transgender rat model is among the first studies to investigate the link between gender-affirming hormone therapy (GAHT) and an adverse condition as hypertension. We demonstrated that estradiol administration in orchiectomized rats resulted in a partial or complete reversal of the deleterious effects caused by testosterone deficiency on bone health. However, despite the novelty of this research, there are notable limitations that should be addressed in future studies. This study does not account for baseline bone health prior to the initiation of GAHT, nor does it model potential confounding factors such as vitamin D deficiency or low physical activity. We chose to use rats with hypertension, a factor capable of reducing BMD, to better approximate clinical conditions and to allow us to simulate real-world scenarios. Investigating the effects of estrogen over a longer period, with and without physical activity, would provide valuable insights. Additionally, since GAHT is typically initiated during puberty and continues into older age, assessing the effects of therapy in animals at different life stages would be beneficial for understanding age-related differences in response.

## Figures and Tables

**Table 1:** Primer sequences for PCR.

Marker	Genebank Code	Product Size (bp)		Sequence
**Housekeeping gene**				
***β-actin***	NM_031144.3	345	Fw	CCTTCAACACCCCAGCCAT
			Rv	CATAGCTCTTCTCCAGGGA
**Markers evaluated for osteogenic differentiation**				
***Col1a1***	NM_053304.1	651	Fw	GCATACACAATGGCCTAA
			Rv	CTGTTCCAGGCAATCCAC
***Osteocalcin***	NM_13414.1	156	Fw	CAAAGCCTTCATGTCCAA
			Rv	TCTGATAGCTCGTCACAA
***RANK-L***	NM_057149.1	432	Fw	TCGCTCTGTTCCTGTACTTT
			Rv	CCCTTAGTTTTCCGTTGCTT
***OPG***	U94330.1	408	Fw	CTCCTGGCACCTACCTAA
			Rv	GTGTTGCATTTCCTTTCTGA

## Data Availability

The data will be available in the CONICET Repository, Argentina.

## Abbreviations

The following abbreviations are used in this manuscript:

ALP	alkaline phosphatase activity
BMD	bone mineral density
BMPCs	bone marrow progenitor cells
CFU-GM	Colony-forming unit–granulocyte macrophage
Col 1a1	collagen type 1 alpha 1 chain
Dy	yield point displacement
Eabs	energy absorbed
ERβ	estrogen receptor β
Es	estrogen
Fmax	maximun load
Fy	load in yield point
GAHT	gender-affirming hormone therapy
GnRH	gonadotropin-releasing hormone
Ix	second moment of inertia of cortical bone
Ob	osteoblast
OC	osteocalcin
OPG	osteoprotegerin
Orch	orchidectomy
Ot	osteocyte
RANK	receptor activator of nuclear factor-κβ
RANK-L	receptor activator of nuclear factor-κβ ligand
WHO	World Health Organization
μCT	micro-computed tomography
